# Chronic wasting disease in Europe: new strains on the horizon

**DOI:** 10.1186/s13028-021-00606-x

**Published:** 2021-11-25

**Authors:** Michael Andreas Tranulis, Dolores Gavier-Widén, Jørn Våge, Maria Nöremark, Sirkka-Liisa Korpenfelt, Maria Hautaniemi, Laura Pirisinu, Romolo Nonno, Sylvie Lafond Benestad

**Affiliations:** 1 Department of Preclinical Sciences and Pathology, Faculty of Veterinary Medicine, Norwegian University of Life Sciences, Post Box 5003, Ås, Norway; 2 National Veterinary Institute (SVA), 75189 Uppsala, Sweden; 3 Department of Biomedical Sciences and Veterinary Public Health, Swedish University of Agricultural Sciences (SLU), Box 7028, 75007 Uppsala, Sweden; 4 Norwegian Veterinary Institute, Post Box 64, 1431 Ås, Norway; 5 Finnish Food Authority, Virology Unit, 00790 Helsinki, Finland; 6 Department of Food Safety, Nutrition and Veterinary Public Health, Istituto Superiore Di Sanità, 00161 Rome, Italy

**Keywords:** CWD, Deer, Fennoscandia, Moose, Nordic countries, Prion disease, Red deer, Reindeer

## Abstract

Prion diseases are fatal neurodegenerative disorders with known natural occurrence in humans and a few other mammalian species. The diseases are experimentally transmissible, and the agent is derived from the host-encoded cellular prion protein (PrP^C^), which is misfolded into a pathogenic conformer, designated PrP^Sc^ (scrapie). Aggregates of PrP^Sc^ molecules, constitute proteinaceous infectious particles, known as prions. Classical scrapie in sheep and goats and chronic wasting disease (CWD) in cervids are known to be infectious under natural conditions. In CWD, infected animals can shed prions via bodily excretions, allowing direct host-to-host transmission or indirectly via prion-contaminated environments. The robustness of prions means that transmission via the latter route can be highly successful and has meant that limiting the spread of CWD has proven difficult. In 2016, CWD was diagnosed for the first time in Europe, in reindeer (*Rangifer tarandus*) and European moose (*Alces alces*). Both were diagnosed in Norway, and, subsequently, more cases were detected in a semi-isolated wild reindeer population in the Nordfjella area, in which the first case was identified. This population was culled, and all reindeer (approximately 2400) were tested for CWD; 18 positive animals, in addition to the first diagnosed case, were found. After two years and around 25,900 negative tests from reindeer (about 6500 from wild and 19,400 from semi-domesticated) in Norway, a new case was diagnosed in a wild reindeer buck on Hardangervidda, south of the Nordfjella area, in 2020. Further cases of CWD were also identified in moose, with a total of eight in Norway, four in Sweden, and two cases in Finland. The mean age of these cases is 14.7 years, and the pathological features are different from North American CWD and from the Norwegian reindeer cases, resembling atypical prion diseases such as Nor98/atypical scrapie and H- and L-forms of BSE. In this review, these moose cases are referred to as atypical CWD. In addition, two cases were diagnosed in red deer (*Cervus elaphus*) in Norway. The emergence of CWD in Europe is a threat to European cervid populations, and, potentially, a food-safety challenge, calling for a swift, evidence-based response. Here, we review data on surveillance, epidemiology, and disease characteristics, including prion strain features of the newly identified European CWD agents.

## Background

Chronic wasting disease (CWD) is a fatal neurodegenerative disease affecting species of the *Cervidae* family. It is a prion disease, of which human Creutzfeldt-Jakob disease, scrapie in sheep and goats, and bovine spongiform encephalopathy (BSE) in cattle are well known [1]. In these maladies, the host-encoded cellular prion protein (PrP^C^) is misfolded into an aggregation-prone conformer called PrP^Sc^ (scrapie), aggregates of which constitute transmissible prions [2, 3]. Misfolded PrP conformers interact with PrP^C^ molecules and convert these into three-dimensional copies of the misfolded conformer, and these can have neurotoxic effects. For typical prion-induced neurotoxicity to occur, PrP^C^ (i.e., the substrate for prion propagation), must be present at the neuronal cell surface [4, 5] to which it is normally attached with a glycosylphosphatidylinositol (GPI) anchor [6]. Animals without PrP^C^ are completely resistant to prion infection [5, 7]. Investigations of animals devoid of PrP^C^, either transgenic [8] or naturally occurring [9], have identified a physiological role for PrP^C^ in maintenance of peripheral nerve myelin.

Misfolding of PrP^C^ can occur spontaneously or be caused by somatic or germ-line mutations in the gene encoding PrP^C^, giving rise to sporadic or inherited prion diseases [10]. Exposure to exogenous prions, either through experimental challenge, iatrogenic or natural infection, can also induce prion disease. Indeed, the transmissibility of prion diseases was recognized early, and the diseases were called transmissible spongiform encephalopathies (TSE) [11]. Although, uniformly transmissible, only classical scrapie in small ruminants [12], CWD in cervids [13, 14], and the more recently discovered prion disease of dromedary camels [15], can transmit horizontally under natural circumstances. A commonality of the infectious and environmentally transmissible prion diseases is the accumulation of prions in peripheral tissues, most notably lymphoid organs, including those along the gastro-intestinal tract, facilitating the release of prions in excretions [16–21]. Although prion infectivity has been demonstrated in peripheral tissues, including in sporadic prion diseases in both humans [22] and atypical prion diseases in animals [23], prion release to the surroundings does not normally appear to reach the levels needed for horizontal transmission. The distinction between sporadic and infectious disease aetiology is fundamentally important in assessment and management of animal prion diseases. This has been demonstrated most clearly in classical versus Nor98/atypical scrapie, with the former transmitting horizontally between genetically susceptible animals and the latter occurring sporadically in old animals [24, 25]. Whilst infectious classical scrapie has been effectively combatted by stamping out, imposing limitations on animal movements, and, importantly, by selective breeding for increased resistance [26], such efforts have had minimal impact on the sporadic occurrence of Nor98/atypical scrapie.

CWD, as characterized from North America during the last 50 years, has clearly presented as an infectious disease, transmitting horizontally within and between several cervid species, in many instances with similar efficiency as that of classical scrapie in sheep [13]. Accordingly, the presence of CWD prions has been observed to be prominent in peripheral tissues, and the release of CWD infectivity in saliva [27, 28], urine [29, 30], and faeces [31] has been documented even at preclinical stages. Outbreaks of CWD caused by prion-contaminated environments have also occurred [32–36] and indirect transmission through the environment may, in heavily contaminated areas, be the main mode of transmission. CWD has spread relentlessly in North America and is currently present in 26 US states and three Canadian provinces [37], affecting both farmed and free-ranging deer, particularly white-tailed deer (*Odocoileus virginianus*), mule deer (*Odocoileus hemionus*), and rocky mountain elk (*Cervus canadensis nelsoni*). A few cases in North American moose (*Alces alces shirasi*) have also been recorded [38], and a single case has been diagnosed in a captive reindeer (*Rangifer tarandus*) in Northern Illinois [39].

Prions come in varieties known as strains, all of which consist of misfolded and aggregated PrP, but folded and arranged differently [40–47]. The differences may be structurally subtle, but nevertheless clinically and epidemiologically significant, affecting the species transmission spectrum and disease manifestation. Prions are characterized at many levels: by primary host, organ distribution, and pattern of PrP^Sc^ aggregates, by biochemical characterisation of PrP^Sc^ and, importantly, in bioassays in defined rodent models, such as bank voles (*Myodes glareolus*) [48, 49], and transgenic mice [50–52]. Initial identification and detailed characterization of prion strains depend on bioassays. When transmitting a primary isolate to a rodent model, the attack rate and incubation period are recorded. Furthermore, the pathology and biochemical properties of PrP^Sc^ as revealed by western blot (WB) i.e., glycoprofile (the abundance of glycosylated forms) and fragment size of PrP^Sc^ following treatment with proteinase K, provide valuable information about the strain [53]. The proteinase K resistant fragments of PrP^Sc^ analysed in WB are referred to as PrP^res^ (resistant) in this paper. Upon rounds of sub-passages in the rodent model, the prion strain will adapt to its new host and attack rates will often reach 100%. Simultaneously, the incubation period will drop and stabilize to a level that is reproducible and characteristic for the given strain. Thus, in contrast to bioassays of some other pathogens, such as viruses or bacteria, bioassays of prions change the pathogen itself both conformationally and functionally; some features are lost, whereas others are conserved and/or gained in the process [40]. Although, tedious and imprecise compared with modern nucleic acid-based molecular typing of pathogens, bioassays in well-defined rodent models are currently the best tools available for prion strain characterization. The molecular mechanisms and dynamics concerning host–pathogen interactions are also incompletely understood for prions. For instance, mechanisms dictating tissue tropism of prions, or their potential to undergo divergent or convergent evolution, remain largely unknown.

The first reports comparing European CWD isolates from wild reindeer [54] and moose have now been published [55, 56], showing that these differ from each other and from North American CWD strains. Further, data suggest that sub-strains might be present among the European moose CWD cases [55].

The purpose of this review is to provide an update of the current situation with emergence of CWD in Northern Europe, and, specifically, to address the issue of novel CWD strains and atypical variants of the disease.

### Search strategy

This critical review is based on searches in databases PubMed (https://pubmed.ncbi.nlm.nih.gov/) using the terms; “chronic wasting disease” [Title/Abstract] AND/OR “CWD” AND/OR “Norway” AND/OR “Europe”, “Sweden”, “Nordic countries”. Abstracts of articles identified were evaluated for relevance to the topic. Scientific opinions and governmental advice from European and national working groups also include comprehensive literature searches and evaluations, and these were also a source of information. Data from EU, Norwegian, Swedish, and Finnish surveillance databases and diagnostic reports from relevant authorities have been retrieved. Finally, some unpublished results from ongoing investigations have been made available through personal communication.

### CWD surveillance in Fennoscandia before and after first detection in 2016

#### Norway

Data on surveillance for CWD in Norway have since 2005 been reported in annual reports [57]. Prior to detection of CWD in 2016 [54], 2159 cervids had been tested in Norway. Since only ten of these were wild reindeer, efforts were made to increase sampling of wild reindeer populations. Upon detection of CWD in reindeer and moose, surveillance was intensified [58–60], reaching 127,216 Norwegian cervids tested by March 2021 (Table 1) [61].

**Table 1:** CWD surveillance and diagnoses in Norway (2002–2021)

	2002–15	2016	2017	2018	2019	2020	2021^a^	Sum	CWD
Moose (*Alces alces*)	142	4403 (**2**)	5468 (**1**)	6705 (**1**)	5935 (**2**)	6200 (**1**)	290 (**1**)	**29,143**	**8**
Reindeer (*Rangifer t. tarandus*)									
Semi-domesticated	966	1739	10,940	12,046	12,937	6512	1791	**46,931**	
Wild	10	842 (**4**)	2922 (**9**)	3650 (**6**)	3334	3213 (**1**)	26	**13,997**	**20**
Red deer (*Cervus elaphus*)									
Farmed	825^b^	129	444	643	571	823	118	**3553**	
Wild		2453	3639 (**1**)	7785	5187	3450	161 (**1**)	**22,675**	**2**
Fallow deer (*Dama dama*)	13	15	20	48	37	92	11	**236**	
Roe deer (*Capreolus capreolus*)	203	484	1955	2124	1692	1832	448	**8738**	
Cervid spp.^2^		82	271	655	454	406	65	**1933**	

CWD positive diagnosis is given as number in bold in parenthesis and the total number of tested animals and the total number of diagnosed cases are also prenseted in bold in the two rightmost columns of the tables

^a^April 1st

^b^Red deer was pooled wild and farmed for the years 2002–2015

^2^Analysis of cervid samples, but species not given at the time of analysis. Positive diagnoses of CWD in parentheses

#### Sweden

With the exception of EU-regulated active surveillance 2007–2010 [62], surveillance of CWD in Sweden was passive until 2016, based on reporting and examination of animals with clinical signs. From 2016, routine sampling of cervids submitted for necropsy was introduced and archived frozen brain samples that had been collected between 2008 and 2016 were examined retrospectively [63]. From 2018, EU-regulated surveillance was introduced, focusing on cervids displaying symptoms, found dead, road killed or euthanized. The surveillance also includes slaughtered or hunted cervids found unfit for human consumption. After detection of positive cases in moose, increased surveillance in areas surrounding the positive cases has been performed, and has included healthy animals (slaughtered or hunted) found fit for human consumption [64]. Results of the surveillance are shown in Table 2.

**Table 2:** CWD surveillance and diagnoses in Sweden (2007–2021)

	2007–15^b^	2016	2017	2018	2019	2020	2021^a^	Sum	CWD
Moose (*Alces alces*)	171	74	192	157	857 (**3**)	249 (**1**)	21	**1721**	**4**
Reindeer (*Rangifer t. tarandus*)^c^		2	21	15	1965	991	107	**3101**	
Red deer (*Cervus elaphus*)	1							**1**	
Farmed				5	29	82	11	**127**	
Wild		6	6	8	2	2		**24**	
Fallow deer (*Dama dama*)	1		8			1		**10**	
Roe deer (*Capreolus capreolus*)	96	14	13	15	73	71	16	**298**	
Cervid spp.	195^d^							**195**	

CWD positive diagnosis is given as number in bold in parenthesis and the total number of tested animals and the total number of diagnosed cases are also prenseted in bold in the two rightmost columns of the tables

^a^March 25th

^b^Including 270 samples examined retrospectively in 2016

^c^All semi-domesticated

^d^Species known at the time of analysis, but not published in reports. Positive diagnoses of CWD in parentheses

#### Finland

The occurrence of CWD in wild cervids and reindeer has been monitored in Finland since 2003. The white-tailed deer population in Finland, app. 125,000 animals, originates from North America, which is why Finland targeted CWD surveillance especially to white-tailed deer during the EU-regulated surveillance in 2007–2010. From 2018, EU-regulated CWD surveillance in Finland has targeted clinically ill, fallen/road or predator killed cervids. Slaughtered or hunted cervids considered unfit for human consumption by an official veterinarian, are also sampled. Increased surveillance has been performed in areas surrounding the positive cases in 2018 and 2020, and this has also included hunted animals. A summary of CWD surveillance in Finland is given in Table 3.

**Table 3:** CWD surveillance and diagnoses in Finland (2006–2020)

	2006–10	2011–16	2017	2018	2019	2020	2021^a^	Sum	CWD
Moose (*Alces alces*)	86	51	48	242 (**1**)	162	200 (**1**)	29	**818**	**2**
Reindeer (*Rangifer t. tarandus*)^b^	45	29	16	294	616	624	95	**1719**	
Finnish forest reindeer (*Rangifer t. fennicus*)^c^	3	4	13	14	12	7	8	**61**	
White-tailed deer (*Odocoileus virginianus*)^d^	612	27	23	50	131	125	25	**993**	
Red deer (*Cervus elaphus*)						1		**1**	
Fallow deer (*Dama dama*)	10	3	1					**14**	
Roe deer (*Capreolus capreolus*)	23	14	13	63	208	255	47	**623**	

CWD positive diagnosis is given as number in bold in parenthesis and the total number of tested animals and the total number of diagnosed cases are also prenseted in bold in the two rightmost columns of the tables

^a^April 15th

^b^All semi-domesticated

^c,d^Wild. Positive diagnosis of CWD in parentheses

#### The EU programmes 2007–2010 and 2018–2020

Between 2007 and 2010, an EU-regulated surveillance of CWD was performed in Europe [62]. The results were evaluated by the European Food Safety Authority (EFSA) in 2010, and it was concluded that the presence of CWD could not be excluded [65]. One shortcoming of the surveillance programme that was identified was that sampling had mainly focused on healthy animals [66] and had tested a low number of cervids (600 per country). After detection of CWD in Norway, EFSA was asked to assess the need for surveillance for CWD in Europe. Based on the scientific opinion from EFSA [67] that suggested a surveillance programme with the aim of confirming or excluding the presence of CWD in countries where the disease had not been detected, and estimation of the prevalence and geographical spread, a three-year surveillance programme was initiated through European Commission regulation [68]. This programme covers all EU member states with populations of moose and/or reindeer, i.e., Estonia, Finland, Latvia, Lithuania, Poland, and Sweden. Species included in the surveillance were Eurasian tundra reindeer, Finnish forest reindeer (*Rangifer tarandus fennicus*), moose, roe deer (*Capreolus capreolus*), white-tailed deer (*Odocoileus virginianus*), and red deer. The sampling target was set to 3000 captive cervids and 3000 wild or semi-domesticated cervids, but to be adapted based on population size. In addition, sampling should focus on fallen/culled, clinically sick, and road- or predator-killed animals, or hunted or slaughtered cervids which have been declared unfit for human consumption by meat inspection. Brainstem and lymph nodes should be sampled and analysed by one of the rapid tests approved in the regulation [68], and positive results confirmed by WB or immunohistochemistry (IHC). In case of confirmed-positive cases, the sampling should be increased based on a risk assessment. The results of this programme are reported to EFSA by the member states involved and published in annual reports [69]. Although sampling animals of the selected target groups could be challenging due to the necessary logistics when animals die in remote areas [70], EFSA concluded in 2019 [71] that testing higher risk animals is more informative about the disease than testing healthy animals [71]. Funding and political will have had an impact on the number of samples collected in different countries [70].

### Outbreak in Norwegian wild reindeer—management and disease features

**Fig. 1:**
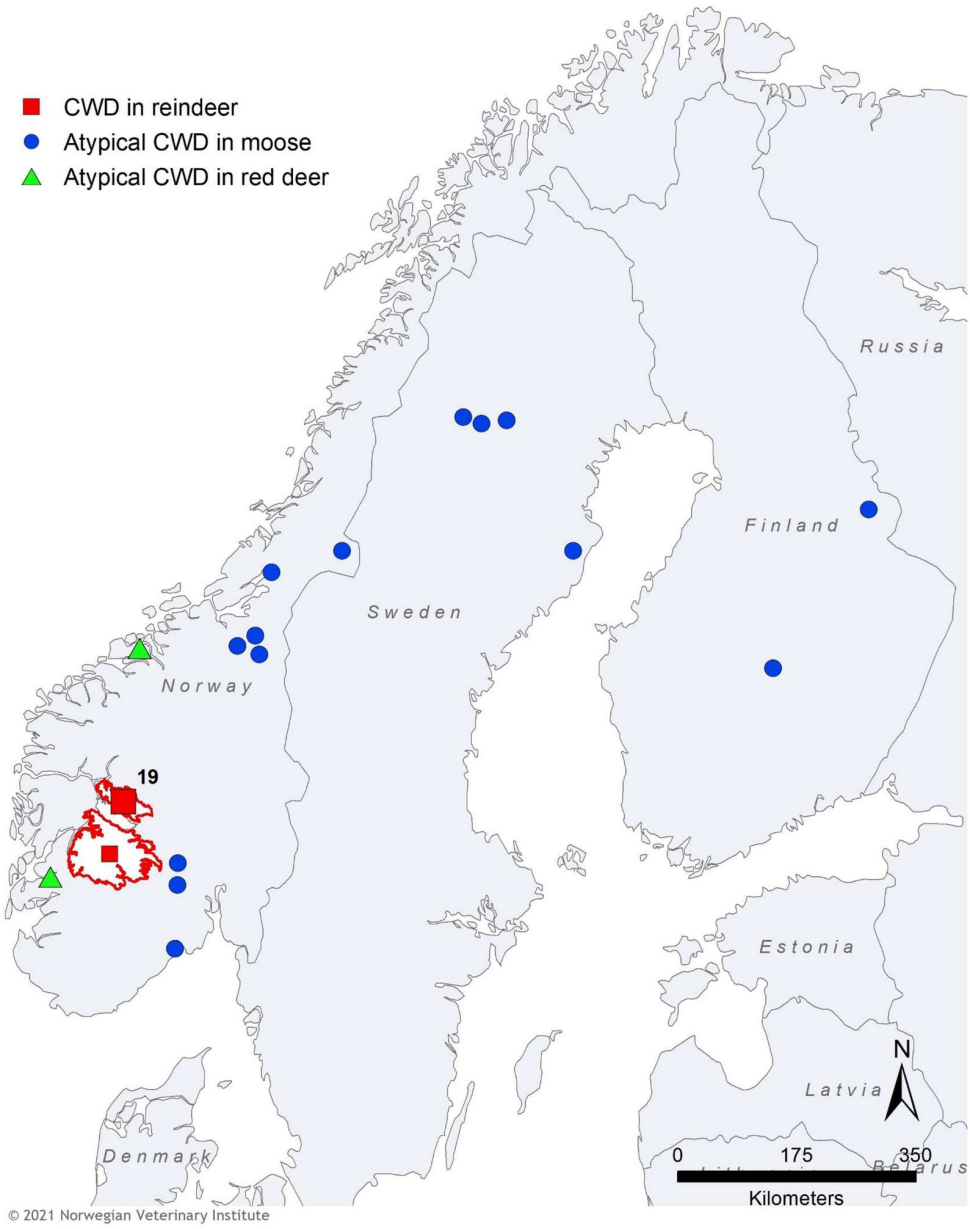
Distribution of CWD in reindeer, red deer, and moose in Fennoscandia as per April 2021. The outbreak of CWD among reindeer in Nordfjella, with a total of 19 cases, is indicated with a red square. The subsequent case diagnosed in a reindeer in 2020 is indicated with a smaller red square. The Nordfjella and Hardangervidda reindeer areas are marked with a red line. Atypical cases of CWD in moose are shown with blue circles and the two cases in red deer with green triangles

In March 2016, a team from the Norwegian Institute for Nature Research observed a diseased reindeer during field operations (collaring) in the Nordfjella wild reindeer population (Fig. 1). It died after a few minutes and was submitted to the Norwegian Veterinary Institute for necropsy. After the diagnosis of this first CWD case, a surveillance programme was launched, resulting in the identification of three additional positive reindeer during the 2016 hunting season.

**Fig. 2:**
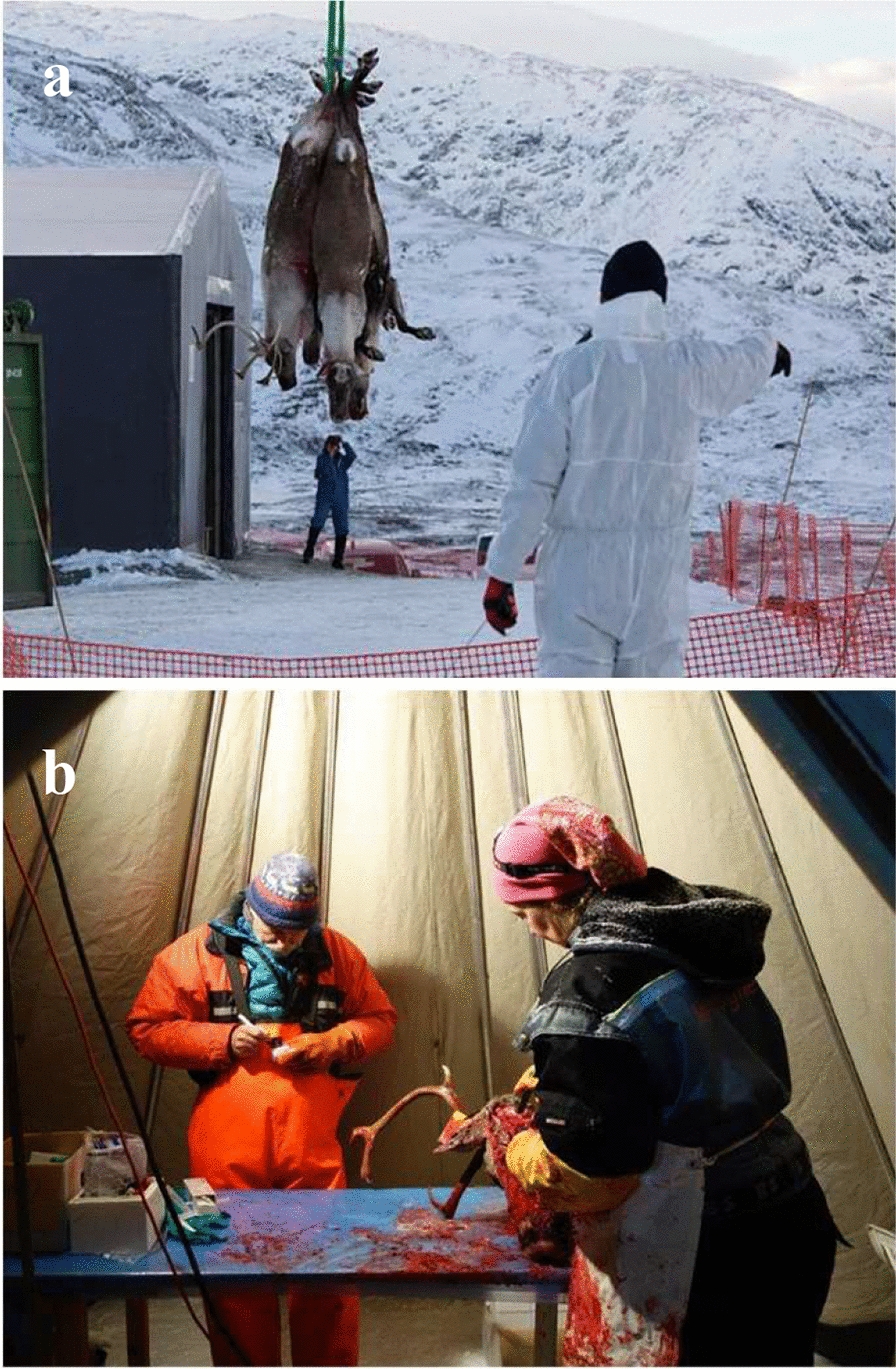
Images from the cull of the reindeer population in Nordfjella. Shot animals were transported with snowmobiles or helicopter to sites for sampling. All carcasses were destroyed with incineration after sampling

The Norwegian Food Safety Authority and the Norwegian Environment Agency requested the Norwegian Scientific Committee for Food and Environment for a scientific opinion to address food-safety aspects of CWD and management options concerning the outbreak of contagious CWD among wild reindeer in the Nordfjella area. This opinion concluded that eradication of CWD in wild reindeer in Norway, could only be achieved through culling the affected population [72]. Surveillance data prior to, and during, the cull confirmed a low prevalence, and animal-to-animal spread was considered the predominant mode of transmission, rather than via environmental contamination [73]. However, the Nordfjella area is actively used as summer pastures for sheep (around 50,000 per season) and a high number of salt licks for sheep are allocated in the area, several of which were also used by wild reindeer. Environmental build-up of CWD infectivity at such “hot spots” was therefore considered a high risk [73]. Importantly, despite neighbouring populations of semi-domesticated reindeer to the north, wild reindeer to the south, and sympatric red deer and moose, the affected population was considered to be semi-isolated from these by roads and railway. Considering all these aspects, culling of all reindeer in the affected area was advised (Fig. 2). A fallow period of a least five years was recommended before reintroduction of reindeer to the area.

Data from North America have shown that mule deer [74] and white-tail deer [75] males are more likely than females to test positive for CWD. The results from > 2000 culled Norwegian wild reindeer revealed a similar distribution [76]. The chance of positive CWD-diagnosis for adult males was about 2.7 times (P < 0.05) the risk of females. This knowledge, together with other disease characteristics, were used when modelling was refined to assist hunting and management strategies [77, 78]. Norwegian wild reindeer and other wild cervid populations are regulated by recreational hunting, making this important for surveillance and disease management [79].

Genetic variation in *PRNP*, the gene encoding cellular PrP, has long been recognized as an important factor in prion-disease modulation in both animals and humans. Prior to detection of CWD in Europe, characterization of *PRNP* genetic variation among European cervids was limited to studies of non-affected populations [80, 81] and later, [82, 83] studies of genetic variation across endemic species. Culling of the Norwegian wild-reindeer population affected with CWD [84] allowed correlation of *PRNP* variation with diagnosis of CWD [85]. Five variants (alleles) of the *PRNP* gene (A–E) were identified in Norwegian reindeer, combining into 14 *PRNP* genotypes. Two of the alleles, A, thought to be the archetypic gene variant in reindeer, and C (octapeptide deletion) were both strongly overrepresented (P < 0.001 for animals carrying two copies of the A or C allele) among the CWD animals, whereas the opposite was the case for animals carrying the B allele in which serine in position 225 is substituted with tyrosine. Despite small numbers, the authors concluded that the *PRNP* genetic influence on susceptibility of reindeer to the CWD strain observed in Norwegian reindeer is substantial [85]. These data are valuable for further *PRNP* genetic susceptibility-profiling of non-affected wild and semi-domesticated reindeer populations.

## CWD in Norwegian reindeer

**Fig. 3:**
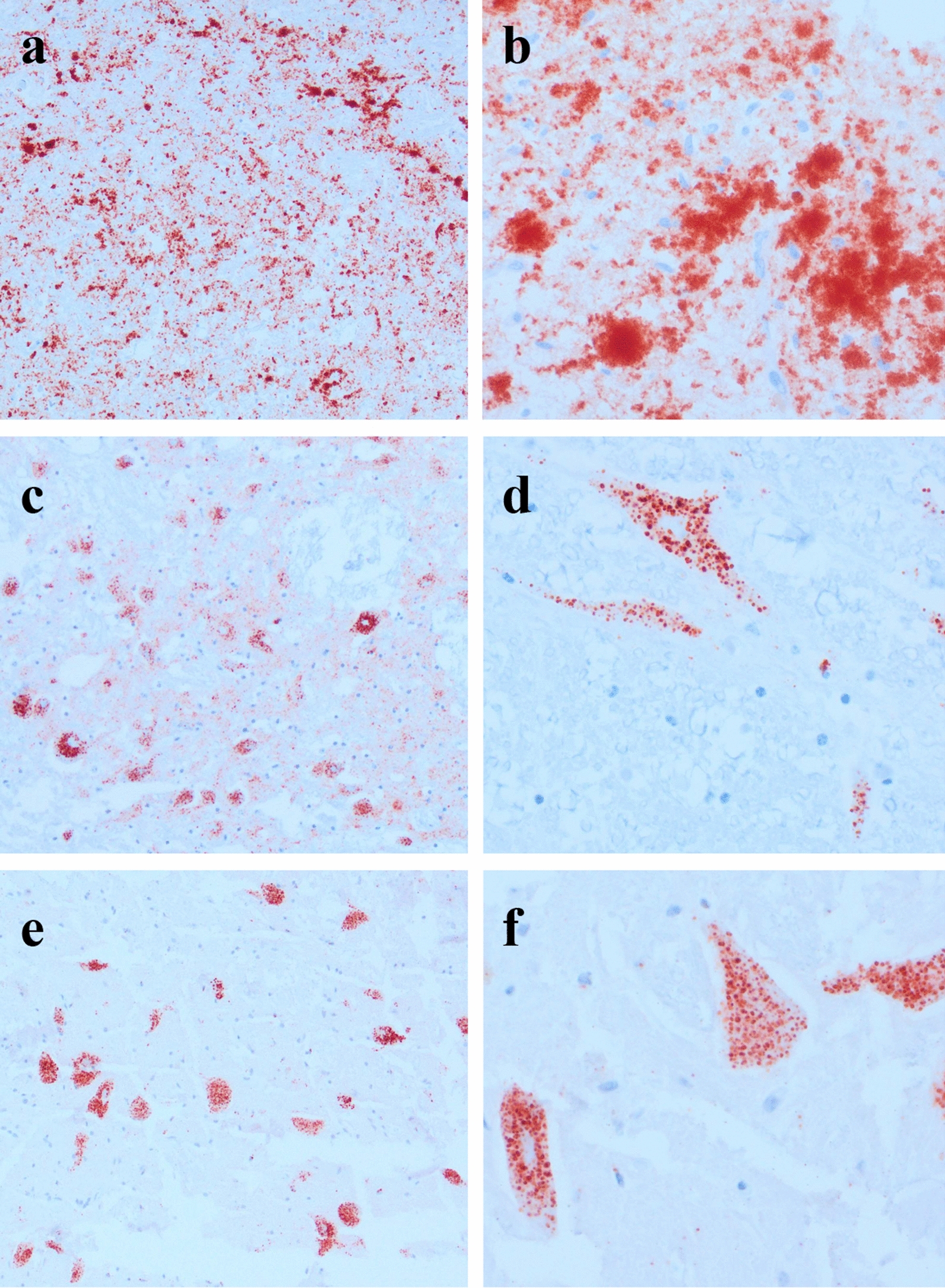
Immunohistochemical labelling of PrP^Sc^ in the brain of reindeer and moose diagnosed with CWD. **a**, **b** Reindeer, medulla oblongata, strong, extracellular thin and coarse granular, coalescing and plaque-like PrP^Sc^ deposition (SAF84 mAb). **c**, **d** Atypical CWD in a Norwegian moose, medulla oblongata, predominance of intraneuronal PrP^Sc^ deposition (L42 mAb). **e**, **f** Atypical CWD in a Swedish moose, thalamus, intraneuronal PrP^Sc^ deposition (SAF84 mAb)

The first case of CWD in reindeer was detected by testing for the presence of PrP^Sc^ on medulla oblongata at the level of the obex with an enzyme-linked immunosorbent assay (TeSeE SAP ELISA CWD, Bio-Rad) (Fig. 3a, b). As a confirmatory method, WB (TeSeE, Bio-Rad) was applied. Three proteinase K-resistant bands with estimated molecular mass between 17 and 29 kDa were obtained from the medulla sample. This three-banded pattern was indistinguishable from that of a North American CWD isolate [54], thus providing no hints of the strain differences that would later appear from transmission experiments.

Visualisation by IHC of PrP^Sc^ deposits in tissues using monoclonal antibody (mAb) immunolabelling will hereafter be referred to as “staining”. The PrP^Sc^ distribution in this animal resembled what has been described in North American CWD cases, with heavier staining in the ventral brain areas, as extensive perineuronal, perivascular, granular or plaque-like staining [54]. In the investigated lymph nodes, PrP^Sc^ staining was observed in the lymphoid follicles [54].

### CWD in European moose

For CWD screening of moose, ELISA-based rapid tests, TeSeE SAP ELISA CWD (Bio-Rad) and/or IDEXX HerdCheck ELISA were applied to samples from medulla oblongata at the level of the obex, and retropharyngeal lymph nodes, or other lymphoid tissues, when available. In Norway, the four first CWD cases were identified with the Bio-Rad test and the four last with the IDEXX test. All four cases in Sweden were identified with the Bio-Rad test, while the two cases in Finland were identified with the IDEXX test. Lymphoid tissue was available from all the positive moose, except one Norwegian case. PrP^Sc^ was not detected in any of the lymphoid tissues tested in any of the moose [56, 71]. Positive ELISA results on obex tissues were confirmed by WB (TeSeE western blot Bio-Rad).

IHC characterization of PrP^Sc^ accumulation was published for three Norwegian CWD-positive moose [56] and the following paragraph recapitulates the main findings in that report (Fig. 3c, d). Samples from a CWD-positive reindeer from Norway were included for comparison. It appeared that PrP^Sc^ distribution was distinct from that seen in North American CWD cases. The differences can be subtle, as illustrated by use of anti-PrP mAbs raised against different parts of PrP as well as examination of several brain regions and lymphoid tissues. IHC was performed with a panel of five anti-PrP mAbs raised against different parts of PrP. All three moose showed PrP^Sc^ staining in the brain, with the intensity, patterns, and distribution of the staining differing among the animals. PrP^Sc^ was predominantly observed as intraneuronal aggregates and, to a lesser degree, as intra-astrocytic and intra-microglial staining in the cerebral cortices and the olfactory bulb. In the obex area, stained neurons were observed in all nuclei, but unlike observations from most CWD cases, the dorsal motor nucleus of the vagal nerve (DMNV) was not strongly stained. The cerebellum was positive in one of the moose, while PrP^Sc^ staining was observed in all other brain areas in all three animals. Importantly PrP^Sc^ staining was not detected intra-neuronally with the more N-terminal mAbs, in any of the three moose [56]. Furthermore, PrP^Sc^ was not detected in any of the lymphoid tissues of the moose. Overall, these features were different from those observed in reindeer, where PrP^Sc^ distribution was similar to that described in North American cervids with CWD [86–88] and in which the panel of all five mAbs revealed the same PrP^Sc^ accumulation and distribution [56].

The variability of IHC staining properties and WB band patterns among CWD cases in moose is still under investigation and the scientific significance of these observations also remains to be clarified. Preliminary data from IHC characterization of the brain of CWD-positive moose in Sweden and from additional moose in Norway have confirmed a predominance of intraneuronal PrP^Sc^ accumulation (Unpublished observations).

In summary, IHC with the panel of mAbs demonstrated differences in PrP^Sc^ staining between moose and reindeer (Fig. 3e, f). In the first three Norwegian CWD moose analysed, the lack of staining with mAbs that bind to the N-terminal tail of PrP indicate that this part of the protein, in these cases, had been removed by endogenous proteases. The brain distribution and patterns of PrP^Sc^ accumulation in the brain and in the lymphoid system differed between moose and reindeer and, importantly, some differences were observed among the three moose.

**Fig. 4:**
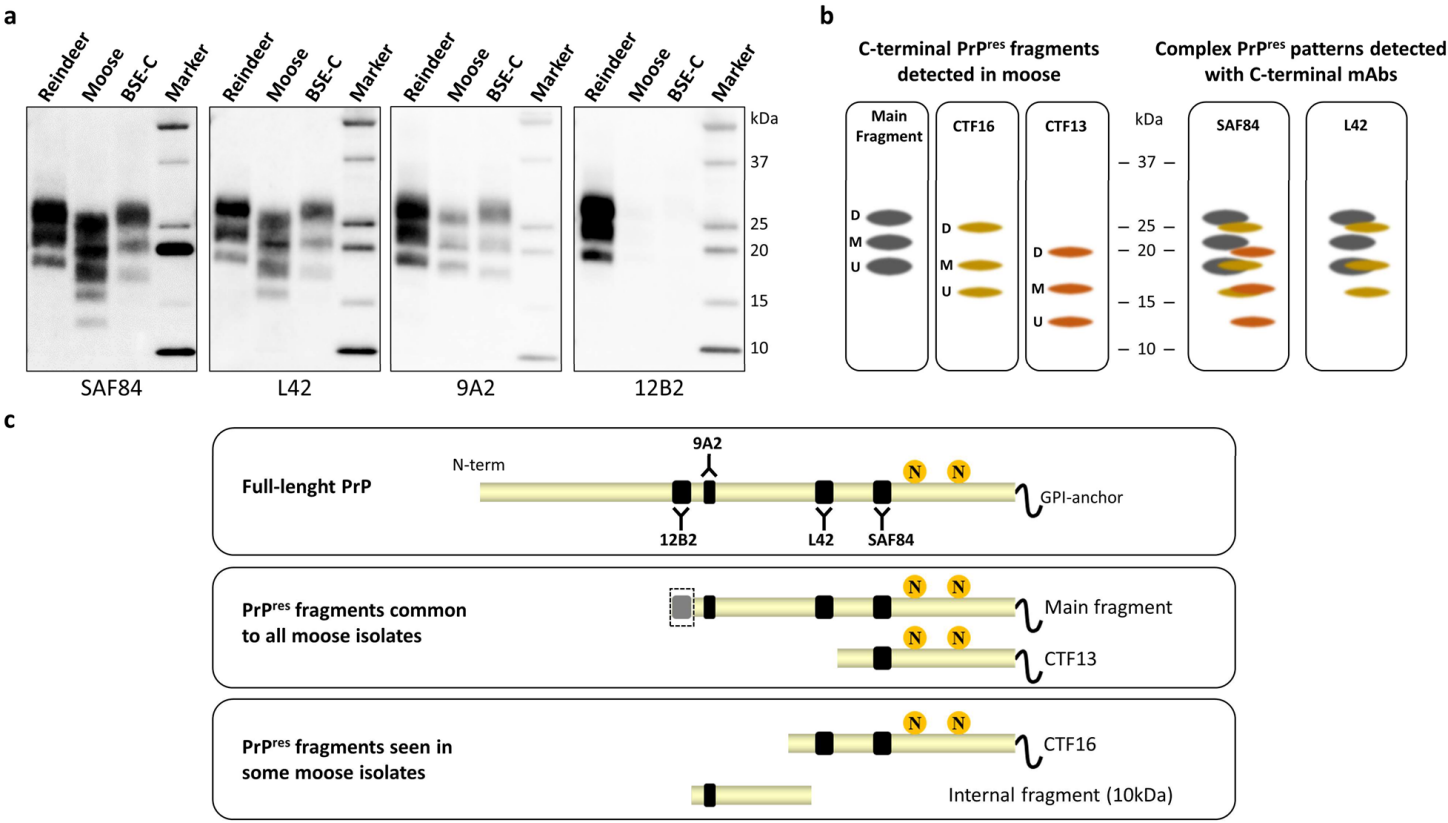
Western blots analysis and interpretation of protease K-treated PrP^Sc^ (PrP^res^) from Norwegian reindeer and moose diagnosed with CWD, and from a bovine with classical BSE (BSE-C). **a** Replica blots analysed with mAbs recognizing different epitopes on PrP, as indicated below each blot. The epitopes of the mAbs are shown in **c** The positions of molecular weight (MW) markers are indicated on the right of the blots (in kilodaltons). Reindeer CWD is detected by the N-terminal mAb 12B2, while moose CWD and BSE-C are not. With mAb 9A2 all samples show a three-banding pattern representing di-, mono- and un-glycosylated PrP^res^, whose molecular weight is lower in moose than in reindeer (“Main fragment” in **b** and **c**). In contrast, mAbs directed to more C-terminal epitopes, L42 and SAF84, recognize additional low molecular weight PrP^res^ fragments at ~ 13 and 16 kDa, detected in moose (referred to as CTF16 and CTF13 in **b** and **c**), which are glycosylated too (51). **b** Cartoons representing PrP^res^ fragments detected in moose with CWD. Main, CTF16, CTF13 fragments are represented separately, with their di-glycosylayed (D), mono-glycosylated (M) and un-glycosylated (U) bands. On the right, the cartoons representing the different PrP^res^ fragments detected in moose PrP^Sc^ are joined to show the interpretation of the complex WB patterns observed with SAF84 and L42. The position of the cartoons is in accord with the position of the bands in the blots shown in a. **c**. Cartoons representing full-length PrP and the different PrP^res^ fragments detected in moose, with the position of the epitopes recognised by the mAbs used in **a** (ovine PrP numbering: SAF84, aa 167–173; L42, aa 148–153; 9A2, aa 99–101; 12B2, aa 93–97). Note that the 12B2 epitope is shaded in the main PrP^res^ fragment to indicate the variability in the N-terminal Proteinase K-cleavage observed in moose with CWD. Yellow circles labelled with “N” represent the position of N-linked sugars. The GPI anchor is represented at the C-terminus

Detailed WB analysis of proteinase K resistant PrP^res^ fragments of the three first Norwegian cases in moose revealed a different banding pattern than that observed in Norwegian reindeer or for Canadian isolates in different cervid species [56]. The PrP^res^ from the Norwegian moose had a lower molecular weight, explained by the removal of larger N-terminal parts of the protein by proteinase K, as confirmed by the partial loss of the 12B2 epitope. This includes residues 93-WGQGG-97 in cervid PrP (Fig. 4A, 9A2 and 12B2 blot). Although the 12B2 epitope was mainly absent for all moose, the amount of PrP^res^ still detectable by 12B2 was variable among the first three moose, suggesting variability in the N-terminal proteinase K-cleavage sites. This variability has also been observed among new cases in moose that are currently under investigation.

Furthermore, by using more C-terminal mAbs, such as SAF84 and L42 (Fig. 4a), it was found that moose PrP^res^ gave an unusual and complex WB pattern that could be explained by the presence and overlapping of additional fragments (Fig. 4). All moose isolates showed the presence of a C-terminal fragment of ≈13 kDa (CTF13) detected by SAF84 (Fig. 4). Importantly, the PrP^res^ pattern varied slightly among the Norwegian moose cases. Indeed, an additional C-terminal fragment of 16 kDa (CTF16) was detected in moose-2 and moose-3 (Fig. 4a, SAF84 and L42 blots). Another fragment of 10 kDa, cleaved by proteinase K at both N and C termini of PrP^Sc^, was detected in moose-1 and moose-3 [56]. The PrP^res^ banding patterns derived from Norwegian moose cases were compared with PrP^res^ features from a Canadian moose case of CWD. In contrast to the Norwegian cases, the Canadian case had PrP^res^ patterns that were indistinguishable from those of other CWD isolates from Canadian cervids. In addition to these molecular observations, cases of CWD in North American cervids with detectable PrP^Sc^ in lymphoid tissues have been reported, which contrasts with findings from CWD isolates from Fennoscandian moose [38, 89]. This suggests that the atypical PrP^Sc^ type observed in Norwegian moose could not simply reflect a factor related to host species.

In order to investigate the possible relationships between CWD in moose and TSEs in other animal species, moose PrP^res^ fragments were also compared with PrP^res^ derived from European sheep and cattle [56]. In these studies, moose PrP^res^ was clearly distinguishable from the most common animal TSEs, such as classical scrapie and atypical scrapie in small ruminants, as well as from classical and atypical BSE in bovines (see, for example, Fig. 4a). Although some similarities were observed with a rare form of scrapie, referred to as CH1641-like, cases of CH1641 scrapie have not yet been detected in Norway [56]. The CH1641 scrapie isolate was derived from a natural case of scrapie in a Cheviot sheep [90] and has attracted interest because of shared molecular features with experimental BSE in sheep [91]. A few natural isolates have been described in sheep in Europe, showing molecular and biological similarities to CH1641 [92–94], and these were named CH1641-like. All cases of CH1641 scrapie reported in EU so far were detected during the surveillance activity on animal TSEs in Europe and no such cases have been recorded in Fennoscandia.

### Confirmed cases in moose—management and disease features

After detection of positive cases in moose, surveillance in areas surrounding the cases was launched in Norway, Finland, and Sweden. The aim was to assess disease occurrence as a basis for control or management options. Moose often live alone and have a complex movement behaviour, with some animals remaining close to their home range, others dispersing, and some travelling larger distances (150–200 km) in a migratory pattern that is sometimes seasonal [95]. As surveillance prior to the detection of positive cases has been very limited, the prevalence and the geographical extension of the disease in those areas where the cases were detected was largely unknown.

As data from surveillance and characterisation of the isolates accumulated, it became clear that the cases had features in common that were distinct from the findings in wild reindeer and from North American cases. The most prominent of these features were the old age of the animals and particular distribution of detectable prions. All cases in moose in Fennoscandia detected so far have been in moose above 10 years of age (Table 4), with prions detected in brain, but samples from lymph nodes being negative. This could suggest that these cases may be of spontaneous, rather than contagious origin [56, 72]. The Norwegian Scientific Committee for Food and Environment evaluated the emerging data in conjunction with established knowledge on moose ecology and concluded that a culling strategy would not be efficient in controlling the disease. However, spatially targeted harvesting, based on epidemiological observations, was also considered a viable option. The majority of the moose are hunted at young age, especially males, resulting in a skewed age structure in the population with few males reaching above 10 years of age, which may be a reason for cases being detected predominantly in female moose [96]. None of the countries that have identified the disease in moose have used a culling strategy so far, and are still undertaking intensified sampling, epidemiological investigations, and further characterisation of the isolates.

**Table 4:** Sex and age-distribution of moose diagnosed with CWD in Fennoscandia

	Sex		Age, years
	Male	Female	
Norway	1	7	12, 13, 13, 13, 14, 15, 17, 20
Sweden		4	10, 14, 16, 16
Finland		2	15, 18
Sum	1	13	Mean age: 14.7

### CWD cases in Norwegian red deer

Two cases of CWD have been observed in wild red deer [97, 98]. The first case was an asymptomatic 16-year-old female shot grazing in farmland during regular hunting in 2017. CWD was diagnosed as described for the moose and reindeer cases. The obex was shown to be positive by ELISA, but all lymph nodes were negative. IHC of obex, lymph nodes, and tonsils was conducted using three different mAbs [54]. PrP^Sc^ deposition was demonstrated in the obex area with two of the mAbs, both showing coarse granular accumulation in the neuropil as well as perineuronal, intraneuronal, and linear staining in several nuclei and axonal tracts. The mAb raised against the N-terminal part of the PrP showed weaker labelling and no intraneuronal deposition. Staining was not shown in any of the lymphoid tissues tested [98]. In September 2021, a further case of CWD in red deer was reported from Norway [97]. Still under investigation, diagnostic data suggest that this case is similar to the first case described above i.e., an atypical form of the disease. The findings from these cases differ from those shown in red deer experimentally infected by the oral route with isolates from North American CWD, and which resulted in a disseminated prion disease with PrP^Sc^ accumulation in the brain, lymphoreticular and other peripheral tissues [99]. CWD has also been reported in a young captive red deer in North America, which also showed PrP^Sc^ in both brain and lymphoid tissues [100]. The analysis of the PrP^Sc^ features of the two Norwegian red deer cases showed an unexpected PrP^res^ pattern, with some similarities to that seen in BSE in cattle. Bioassays are currently ongoing to determine the characteristics of the strain.

### Transmission experiments and strain heterogeneity

Characterising the properties of the emerging CWD prion strains in Europe is of fundamental importance. Understanding their biological properties and behaviour is essential for designing appropriate control and/or eradication measures and for informing policy. Additionally, strain typing is necessary to provide information on the origin and relatedness of the newly detected CWD cases and for determining the potential risks to animals and human health.

A strain typing study involving transmission to bank voles, compared and characterised CWD prion isolates from various cervid species in Canada (elk, white tailed deer and moose) and Norway (reindeer and moose) [55]. In primary transmission, CWD prion isolates from Canada transmitted efficiently to bank voles, in keeping with previous data obtained using CWD isolates from the USA [101]. Despite the similarity of the molecular and pathological phenotypes with North American CWD cases, the transmission of CWD from Norwegian reindeer was very inefficient, suggesting the involvement of a distinct prion strain. Finally, moose CWD isolates from Norway transmitted efficiently, but with longer survival time than North American CWD isolates. These three different transmission patterns were associated with different neuropathological and PrP^Sc^ characteristics in bank voles. Of note is that some of the peculiar features observed in Norwegian moose [56] were preserved after transmission to bank voles, such as cortical involvement, the presence of abundant intraneuronal PrP^Sc^ deposits, and the complex pattern of proteinase K resistant PrP^Sc^ fragments observed by WB. PrP^Sc^ typing with mAbs directed to different PrP epitopes showed the presence of 13 kDa PrP^res^ fragments (CTF13) in bank voles infected with Norwegian moose CWD isolates, but not in those infected with Norwegian reindeer or North American CWD sources [55]. These findings suggest that these neuropathological and biochemical features could be potentially considered as discriminative phenotypes for Norwegian moose CWD.

Adaptation of the CWD isolates in bank voles by further sub-passaging, led to the isolation of four different bank vole-adapted CWD strains: one from the Canadian cases, which was indistinguishable from the vole-adapted strain previously isolated from several US CWD isolates [101], one from Norwegian reindeer, and two from Norwegian moose cases (derived from moose-1 and moose-2) [55]. While both moose-derived CWD strains were characterised by the presence of CTF13 PrP^res^, they were distinguishable by their main PrP^res^ fragments, showing that the slightly different PrP^res^ pattern observed in CWD isolates from moose-1 and moose-2 was preserved after transmission and adaptation in bank voles and was associated with slightly different neuropathological phenotypes. Finally, it was noticed that the adaptation of the moose-2 CWD strain was slower than with the other CWD isolates, possibly suggesting an ongoing competition between different strain components during the adaptation in bank voles. Such interplay between co-existing prion strains is a known phenomenon, also observed for CWD [102, 103]. In keeping with the complex and variable PrP^res^ WB patterns observed in moose CWD cases [56], this finding might suggest a scenario in which different strains could co-exist in moose and can be selectively propagated during experimental transmission in a heterologous species, such as bank voles. The ongoing molecular and biological characterization of moose CWD cases detected in Fennoscandia will help to clarify this issue.

In summary, the strain typing studies in bank voles demonstrated that the CWD prions found in cervids in Norway are distinct from those found in North America. This suggests that the CWD emergence in Norway is probably not linked to recent introduction from North America. Furthermore, CWD in Norwegian cervids was caused by four different strains, two in moose, one in reindeer and one in red deer. Strain typing of the red deer isolate is under investigation but analysis of the PrP^Sc^ glycoprofile strongly indicates that this is a unique strain. These results suggest that interspecies circulation of CWD strains between moose and reindeer in Norway have not occurred, and this is supported by data obtained so far from CWD surveillance in Norway. However, only a subset of CWD isolates from reindeer and moose has been studied so far, and studies of additional CWD cases, detected in moose in Norway, Finland, and Sweden, as well as several reindeer in Norway are ongoing. It is important to characterize CWD strain variations in moose from Fennoscandia, to assess the potential relationships between the diseases in different cervid species, and, importantly, to assess the zoonotic potential of emergent or newly recognised CWD in Europe.

### Perspectives

The discovery of CWD in Norway in 2016 was surprising because previous wildlife disease monitoring and CWD surveillance in Norway and other European countries, even if limited, had not indicated that this disease occurred in Europe. Five years later, the situation has dramatically changed. CWD has been diagnosed in moose in Norway, Sweden, and Finland, in a novel, potentially sporadic, form. The epidemiology, age of the animals, prion strain features, and tissue distribution differ from CWD in its typical contagious form. This, and the diagnosis of yet-another, unique CWD type in a red deer in Norway, shows that CWD no longer is a relatively homogenous disease entity, but covers a family of cervid prion diseases caused by multiple prion strains of unknown origin. Moreover, strain variation seems to be present among moose CWD cases encoding identical *PRNP* genotypes. The root-cause of this strain pleiotropy is incompletely understood.

Following the recent developments, with apparent expansion of the CWD family, disease classifications should be modified to reflect this and there should be appropriate support for governments, stakeholders, and the public in addressing these emerging challenges. This would be similar to the approaches used with atypical BSE (BASE/L-BSE and H-BSE) in cattle and Nor98/ atypical scrapie in sheep and goats. In these cases, intensive surveillance and epidemiological analyses contributed to recognition of different epidemiological patterns followed by targeted control strategies [68].

Sporadic prion diseases would be expected to occur wherever the relevant age-segment of the species is present. Moreover, members of the *Cervidae* family encode prion proteins with shared structural features that appear to allow inter-cervid species prion-disease transmission. Given the global distribution of cervids, together with the probability of spontaneous cases of CWD, it can be speculated that CWD is present in cervid populations outside North America and Fennoscandia. Current evidence from CWD surveillance in Norway and previous experience from the large-scale testing necessary for detection of Nor98/atypical scrapie in sheep [24], suggest that persistent surveillance is required to address this.

Primary passage of transmission experiments into humanized mice (transgenic mice expressing the human *PRNP* gene) with isolates from three of the Norwegian cases (one moose, one reindeer, and one red deer) were reassuringly negative, suggesting a strong transmission barrier to humans, as previously reported for North American CWD [104]. However, further investigations are ongoing, with several Norwegian CWD isolates and different lines of humanized mice.

## Conclusions

The 2018 cull of the Nordfjella wild reindeer removed the only known European cervid population with contagious CWD. A further case with similar disease characteristics was in 2020 diagnosed in a neighbouring management area, questioning the prospects of CWD eradication in wild reindeer in Western Europe. There is no apparent link between CWD in Norwegian reindeer and the cases in Norwegian moose or the two cases in Norwegian red deer. However, investigations of disease characteristics have revealed interesting similarities between the 14 cases of CWD in moose diagnosed in Fennoscandia and atypical prion disease in cattle and small ruminants, with sporadic occurrence in old animals (i.e., an atypical form of CWD). Several lines of evidence point to prion strain variation among the moose cases. Further investigations aiming at clarifying the molecular basis for, and the scientific and epidemiological significance of these observations are ongoing. The origin of CWD in Europe is unknown, but the newly characterised European strains differ significantly from North American strains, perhaps arguing in favour of an *in-situ* origin rather than recent trans-Atlantic transfer.

## Data Availability

Not applicable.
